# Long non-coding RNA human leucocyte antigen complex group-18 HCG18 (HCG18) promoted cell proliferation and migration in head and neck squamous cell carcinoma through cyclin D1-WNT pathway

**DOI:** 10.1080/21655979.2022.2060452

**Published:** 2022-04-07

**Authors:** Bin Mao, Fan Wang, Jingxia Zhang, Qianqian Li, Kai Ying

**Affiliations:** Department of Stomatology, The First People’s Hospital of Yongkang, Yongkang, Zhejiang, China

**Keywords:** HCG18, head and neck squamous cell carcinoma (HNSCC), long non-coding RNA (lncRNA), proliferation and migration, WNT

## Abstract

Emerging evidence has demonstrated that long noncoding RNA (lncRNAs) play a vital role in the development of head and neck squamous cell carcinoma (HNSCC); however, the biological effects and underlying mechanisms of human leukocyte antigen complex group-18 HCG18 (HCG18) have not yet been reported in HNSCC. In this study, we detected the expression of the HCG18 in HNSCC cell lines and patient tissues. We observed that HCG18 was upregulated in HNSCC patient tissues and cell lines. Furthermore, silencing of HCG18 significantly inhibited proliferation, migration, and invasion of HNSCC cells, whereas the opposite effects were detected in the HCG18-overexpressed group. We also found that HCG18 directly binds to the functional protein cyclin D1. Upregulated cyclin D1 reversed the inhibitory effects of HCG18 in HNSCC cell lines and activated the WNT pathway-related proteins (AXIN2, survivin, c-Myc, and β-catenin) simultaneously. Knockdown of cyclin D1 could accelerate the inhibitory effects of HCG18 and decrease the expression of AXIN2, survivin, c-Myc, and β-catenin. This indicated that lncRNA HCG18 might be involved in the tumorigenesis of HNSCC via the cyclin D1-WNT pathway. These results suggest that lncRNA HCG18 could act as a promising prognostic biomarker and potential therapeutic target in HNSCC patients.

## Graphical abstract

**Figure:**
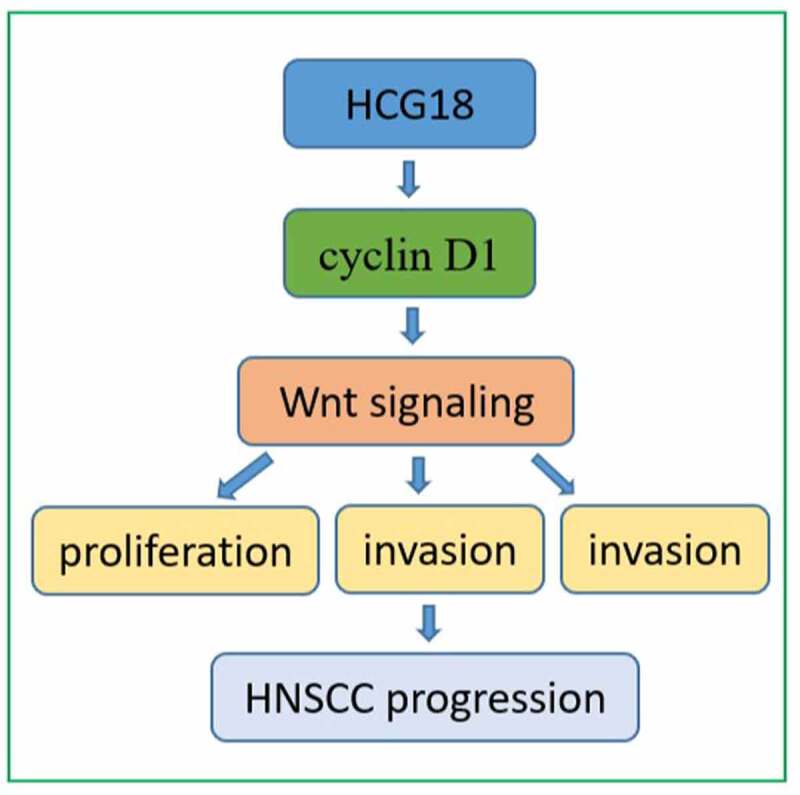

## Introduction

Head and neck squamous cell carcinoma (HNSCC) is the seventh most common malignancy worldwide[1]. Despite tremendous progress have been achieved in the tumorigenesis and treatment of HNSCC, the prognosis of HNSCC patients remains poor[2]. Therefore, a deeper understanding of the biological mechanisms of HNSCC is urgently needed.

Long non-coding RNA (lncRNA) is a type of transcript no longer than 200 nucleotides without protein coding potential[3]. LncRNA has been reported to play a regulatory role by altering the expression level of distinct genes through cis- or trans-acting elements[4]. Increasing evidence has confirmed that multiple lncRNAs exert indispensable effects on the biological process of tumorigenesis and metastasis in distinct types of solid tumors[5]. In HNSCC, several lncRNAs have been identified as potential biomarkers for the diagnosis and prognosis of HNSCC. For instance, lncRNA LINC01296 promotes oral squamous cell carcinoma (OSCC) development by binding to SRSF1, and LINC00662 is reported to promote OSCC growth and metastasis through the miR-144-3p/EZH2 axis[6]. Moreover, LINC01296 and lncRNA HOXA11 have been indicated poor prognosis in HNSCC patients [7,8].

Long non-coding RNA human leukocyte antigen complex group-18 (HCG18) has been reported to promote cell proliferation and metastasis by binding with distinct microRNAs (miRNAs) or functional proteins in several malignancies, such as clear cell renal cell carcinoma and colorectal cancer[9]. However, the regulatory role of HCG18 in HNSCC has rarely been investigated.

Based on the previous studies, we hypothesized that lncRNA HCG18 could facilitate the progression of HNSCC and indicated a poor prognosis. In our study, we aimed to validate the role of HCG28 in the clinical and pathological characteristics of HNSCC, and examine the cellular effects of HCG18 on HNSCC cells. The underlying mechanisms were further explored. Our results provide novel diagnostic biomarkers and therapeutic targets for patients with HNSCC.

## Materials and methods

### The enrolled patients and sample collection

Fifty HNSCC patients who underwent surgery in the Department of Stomatology, First People’s Hospital of Yongkang, from January 2016 to December 2019, were included. None of the enrolled patients had a history of chemotherapy or radiotherapy before surgery. Primary tumors and adjacent normal tissues (at least 2 cm away from the tumor margin) were collected from HNSCC patients within half an hour after surgical specimens were resected, then transferred and stored in liquid nitrogen immediately. The diagnosis of each sample was confirmed by two independent pathologists. This study was approved by the Ethics Committee of the First People’s Hospital of Yongkang.

### Extraction of RNA and quantitative real time polymerase chain reaction (qRT-PCR)

Total lncRNA or mRNA was extracted using the TRIzol reagent ((Invitrogen, Carlsbad, CA, USA). The extracted linear RNAs were reverse-transcribed to complementary DNA, and PCR was conducted using the SYBR Premix Ex Taq Reagent Kit (Takara, Dalian, China) following the manufacturer’s instructions[10]. The results were analyzed using an ABI StepOne Real-Time PCR System (Thermo Fisher, Waltham, MA, USA). GAPDH was used for normalization. The sequences of the primers used were as follows: HCG18 (sense: 5’-ATCCTGCCAATAGATGCTGCTCAC-3’, anti-sense: 5’-AGCCACCTTGGTCTCCAGTCTC-3’), and GAPDH (sense: 5’-CCAGCAAGAGCACAAGAGGAAGAG-3’, anti-sense: 5’-GGTCTACATGGCAACTGTGAGGAG-3’).

### Cell culture

Human HNSCC cell lines (SCC4, SCC25, HN6, HN30, CAL27, and CAL-33) were donated by the Ninth People’s Hospital affiliated with the Shanghai Jiao-Tong University of Medicine. Human oral keratinocytes (HOK), an oral mucosal epithelial cell line, were obtained from gingival tissues of healthy patients. Logarithmically growing cells were cultured in Dulbecco’s modified Eagle’s medium (DMEM) supplemented with 10% fetal bovine serum (FBS) (GIHNSCCO BRL, NY, USA), 100 μg/mL streptomycin, and 100 U/mL penicillin.

### Cell transfection with small interfering RNA (siRNA) or plasmids

Small interfering RNA (siRNA) and overexpressed plasmids were customized by Genomeditech Co., Ltd. (Shanghai, China) and transfected into HNSCC cells using Lipofectamine®3000 (Invitrogen; Thermo Fisher Scientific, Inc.) according to the manufacturer’s instructions[11], and the transfection efficiency was validated by qPCR. Cells were used for further experiments 48 h after transfection.

### Cell proliferation and colony formation assay

The Cell Counting Kit-8 (CCK-8) assay was used to analyze cell proliferation, following the manufacturer’s instructions[12]. Transfected HNSCC and untreated control cells were seeded in 96-well plates and cultured for 24, 48, or 72 h. The optical density was calculated at 450 nm after incubation with 10 μL of CCK-8 for another 2 h. SCC4 and HN30 cells transfected with plasmids or siRNAs of HCG18 were incubated in the plates for 10 days, and colony numbers were counted by staining with crystal violet.

### Cell migration and cell invasion assay

SCC4 and HN30 cells were transfected with si-HCG18, and HCG18-overexpressed plasmids and NC. For the cell migration assay, the cells were suspended and plated in the upper chamber of Transwell plates. DMEM containing 10% FBS was added to the bottom of the chamber. SCC4 and HN30 cells were fixed and stained after incubation for 24 h. The number of cells passing through the membrane was counted in five randomly selected visual fields. For the cell invasion assay, the upper chamber of the Transwell was pre-coated with Matrigel. The cells were then suspended, and the steps described above were followed.

### Western blot experiment

The proteins were lysed using sodium dodecyl sulfate polyacrylamide gel electrophoresis (SDS–PAGE) gels and transferred to a polyvinylidene difluoride (PVDF) membrane (Merck Millipore, Darmstadt, USA) according to the manufacturer’s instructions[13]. After blocking with skim milk, the PVDF membrane was incubated with the corresponding antibodies diluted to 1: 1000. The blotting bands were examined by Odyssey Infrared Imaging System (Biosciences, Lincoln, NE, USA). β-Actin was used as a control protein for normalization. The antibody for cyclin D1 (ab226977), AXIN2 (ab109307), β-catenin (ab68183), c-Myc (ab32072), survivin (ab76424), and β-actin (ab8226) were purchased from Abcam (Cambridge, MA, USA).

### RNA pulldown assay

Biotinylated DNA probes complementary to the fusion site of HCG18 and negative control primer were used for the RNA pulldown assay. SCC4 cells transfected with the HCG18-overexpressed plasmid were fixed, quenched, sonicated, and hybridized. The probes-lncRNA complex was then captured using streptavidin-coated magnetic beads. The probes-lncRNA complexes were eluted and subjected to western blot analysis.

### RNA immunoprecipitation (RIP) assay

The Magna RIP™ RNA-Binding Protein Immunoprecipitation Kit (17–700, Millipore) was used to perform RIP experiments[14]. SCC4 cells were transfected with cyclin D1 overexpressed plasmid or control plasmid and lysed. The lysates from SCC4 cells were incubated with magnetic beads. Magnetic beads were conjugated to human anti-cyclin D1 antibodies or control mouse IgG. The immunoprecipitated RNAs were purified by incubating with proteinase K, the extracted RNA was sent for qPCR, and the enrichment of HCG18 was evaluated.

### Xenograft model construction and in vivo study

HN30 cells were stably transfected with HCG18-overexpressed lentiviral vectors or control vectors. HN30 cells were then injected subcutaneously into the flank of nude mice. Once the xenograft model was formatted, the tumor volume was gauged every 3 days until dissection in the sixth week. The tumor volume was calculated using the following formula: volume (mm^3^) = (L × W [2])/2. All animal procedures were approved by the Animal Ethics Committee of First People’s Hospital of Yongkang.

### Statistical analysis

Functional experiments and PCR data were assessed using Student’s t-test. Relative lncRNA expression levels were determined using the comparative CT method. HCG18 expression levels were divided into high and low levels based on median HCG18 expression. Survival data were evaluated using Kaplan–Meier analysis. Statistical significance was set at P < 0.05. All experimental data were obtained from at least three independent biological replicates and are represented as the mean ± standard deviation.

## Results

Our study aimed to investigate the role of HCG18 in the tumorigenesis of HNSCC. We performed the CCK-8, colony formation, transwell assay and xenograft experiments to validate the cellular effects of HCG18 in vitro and in vivo. Moreover, we conducted RIP assay to explore the interaction between HCG18 and functional protein.

### HCG18 was upregulated in both patients’ tissues and cell lines of HNSCC

**Figure 1.**
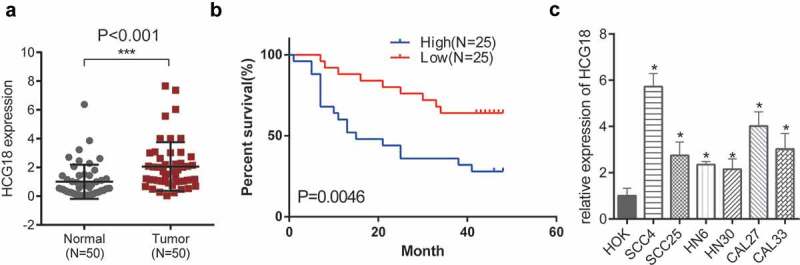
**Expression of HCG18 and its clinical significance in HNSCC**. (a) Expression of HCG18 in HNSCC tissues and adjacent normal tissues (n = 50); (b) OS of HNSCC patients; (c) Expression of HCG18 in HNSCC cell lines (SCC4, SCC25, HN6, HN30, CAL27 and CAL33) and HOK cells; Error bars represent the mean ± SD of triplicate experiments. *P < 0.05; **P < 0.01; ***P < 0.001.

Fifty patients with HNSCC (28 men and 22 women) were enrolled in our study. The median age of the enrolled patients was 62 years. There was no significant relationship between sex (P = 0.573), age (P = 0.982), and the expression of HCG18. We first investigated the expression levels of HCG18 in patients with HNSCC. HCG18 expression was significantly higher in primary tumor tissues of HNSCC patients than in adjacent normal tissues (*P*< 0.001; Figure 1a). Higher HCG18 expression levels in HNSCC patients were correlated with poorer OS (Figure 1b). We observed that HCG18 was upregulated in all HNSCC cell lines examined in this study (SCC4, SCC25, HN6, HN30, CAL27, and CAL-33) compared to that in HOK (Figure 1c). HN30 and SCC-4 cells, which showed the highest and lowest HCG18 expression levels, respectively, were used in the functional experiments. The expression of HCG18 was positively correlated with the tumor stage and grade of lymph node dissemination (*P*< 0.05, Table 1). This indicates that the lncRNA HCG18 is involved in the oncogenesis of HNSCC.

**Table 1.** Relationship between Expression of *HCG18* and clinicopathologic parameters in HNSCC patients

Clinicopathologic parameters	Number of cases	Expression of *HCG18*		*P*-value
		High	Low	
Gender Male Female	28 22	15 10	13 12	0.573
Ages, years ≤59 >59	23 27	11 13	12 14	0.982
T stage, TNM T1+ T2 T3+ T4	26 24	8 17	18 7	0.005
Lymph node metastasis Absent Present	29 21	11 15	18 6	0.021
Alcohol Absent Present	22 28	11 15	11 13	0.804
Smoking Absent Present	21 29	12 15	9 14	0.707
Location Month floor gingival Buccal mucosa Tongue Retromolar	12 17 6 14 1	7 7 3 7 0	5 10 3 7 1	0.782

The stage of TNM was according to the 8th Edition TNM Classification for Head and Neck Cancer. Former/current smokers defined as ≥ one pack-year history of smoking. Positive alcohol use was defined as current alcohol use of ≥ one drink per day for 1 year (12 oz of beer with 5% alcohol, 5 oz of wine with 12% to 15% alcohol, or 1 oz of liquor with 45% to 60% alcohol). All other patients were classified as negative alcohol users.

### HCG18 accelerated the progression of HNSCC cells

**Figure 2.**
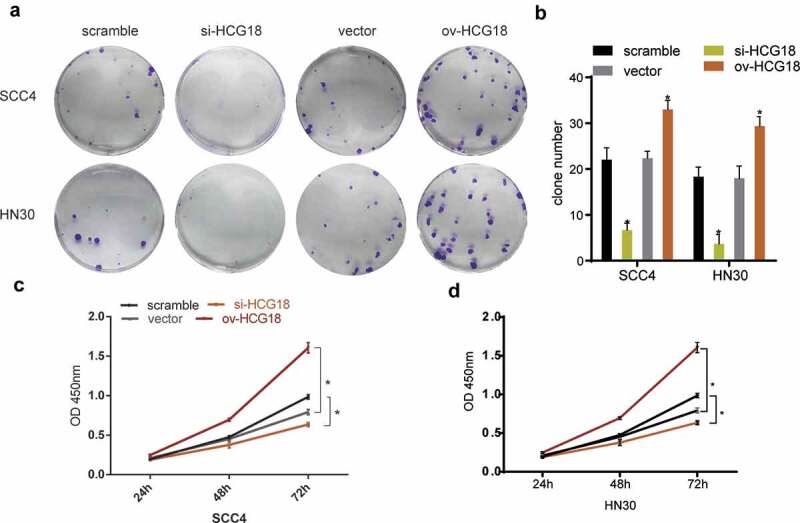
**HCG18 promoted cell proliferation of HNSCC in vitro**. SCC4 and HN30 cells were transfected with silencing HCG18 siRNA (si-HCG18), scrambled siRNA, overexpressed plasmid (ov-HCG18) or vecto*r plasmid* for 48 h, respectively; (a) colony formation assays were performed and representative image was shown; (b) colony number was counted; viability of transfected SCC4 (c) and HN30 (d) cells was accessed by CCK8 assay. Significant differences between groups were analyzed by independent samples t-test. Error bars represent the mean ± SD of triplicate experiments. *P < 0.05; **P < 0.01; ***P < 0.001.

We altered the expression levels of HCG18 to perform functional experiments by transfecting HNSCC cells with siRNAs. The number of clones was lower in HNSCC cells transfected with si-HCG18 than in the negative control (Figure 2(a,b)). The CCK-8 assay showed that low expression of HCG18 downregulated the proliferation ability of HN30 and SCC-4 cells, while the opposite effect was observed by overexpression of HCG18 (Figure 2(c,d)). These findings indicate that HCG18 may facilitate cell proliferation in HNSCC.

### Upregulated HCG18 promoted the invasion and migration of HNSCC

**Figure 3.**
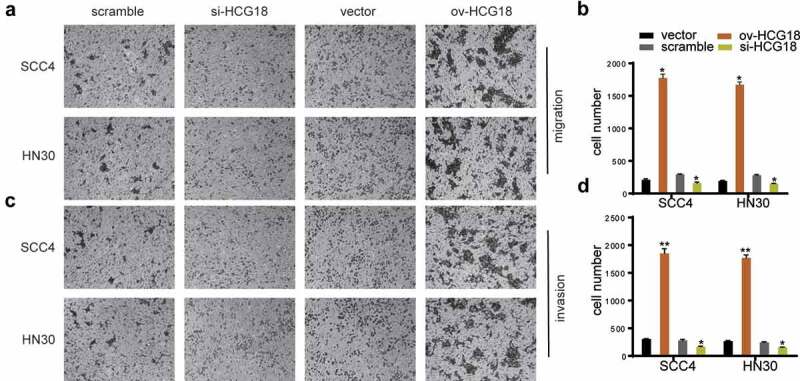
**HCG18 facilitated cell migration and invasion of HNSCC**. SCC4 and HN30 cells were transfected with silencing HCG18 siRNA (si-HCG18), scrambled siRNA, overexpressed plasmid (ov-HCG18) or vecto*r plasmid* for 48 h, respectively; (a) cell migration was measured using transwell assays at 24 h after transfection in SCC4 and HN30 cells respectively; (b) cell number was counted; (c) cell invasion was detected by wound healing assay in SCC4 and HN30 cells, respectively; (d) cell number was counted. Data represent the mean ± SD of at least triplicate independent experiments; *P < 0.05; **P < 0.01; ***P < 0.001.

The invasion and migration abilities of HN30 and SCC-4 cells were significantly inhibited by HCG18 knockdown, whereas the opposite was achieved by upregulating HCG18 (Figure 3(a-d)), indicating that HCG18 could promote the invasion and migration of HNSCC cells.

### HCG18 promoted the tumorigenesis of HNSCC by binding with cyclin D1 and subsequentially regulating the WNT signaling pathway

**Figure 4.**
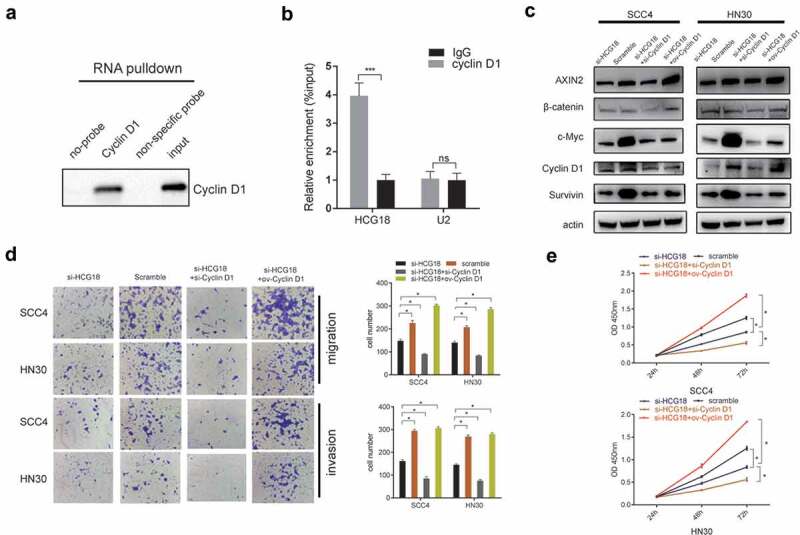
**HCG18 promoted the tumorigenesis of HNSCC by Cyclin D1, and subsequentially activated the WNT signaling**. (a) SCC4 cells was transfected with HCG18 overexpressed plasmid (ov-HCG18) or negative control plasmid, the expression of Cyclin D1 pulled down by HCG18 was detected by western blot assay; SCC4 and HN30 cells were transfected with silencing HCG18 siRNA (si-HCG18), scrambled siRNA, si-HCG18+ silencing Cyclin D1 siRNA (si-Cyclin D1) or si-HCG18+ Cyclin D1 overexpressed plasmid for 48 h, respectively. (b) the expression of AXIN2, β-catenin, c-Myc, Cyclin D1 and Survivin was evaluated. β-actin was used as the internal reference protein for normalization. (c) cell migration and invasion was measured using transwell assays at 24 h after transfection in SCC4 and HN30 cells and cell number was counted respectively; (d) cell number was counted. (D) viability of transfected SCC4 (left panel) and HN30 (right panel) cells was accessed by CCK8 assay. Data represent the mean ± SD of at least triplicate independent experiments; *P < 0.05; **P < 0.01; ***P < 0.001.

A previous study reported that lncRNA HCG18 could stimulate the progression of colorectal cancer cells through the WNT pathway[15]. Herein, we performed an RNA pull-down assay to validate the interaction between HCG18 and cyclin D1, a key functional protein in the WNT pathway. We observed that HCG18 could directly bind to cyclin D1 (Figure 4(a,b)). We also found that AXIN2, c-Myc, survivin, cyclin D1, and β-catenin, which are involved in the WNT pathway, were decreased in HCG18-deficient HN30 and SCC4 cells. Moreover, upregulated cyclin D1 could reverse the inactivation of WNT pathway-related proteins (Figure 4c) and the inhibitory cell effect (Figure 4(d,e)) caused by the knockdown of HCG18 in HN30 and SCC4 cells and vice versa, indicating that HCG18 might promote the tumorigenesis of HNSCC via cyclin D1 and subsequently activate WNT signaling.

### Upregulated HCG18 promoted the progression of HNSCC cells in xenograft model

**Figure 5.**
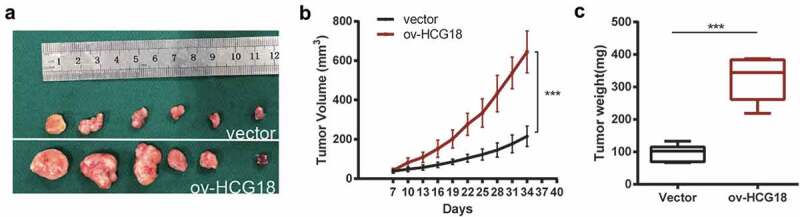
**Upregulated HCG18 promoted the proliferation of HNSCC cells in vivo**. HCG18 transfected cells or control vector of HN30 cells were implanted subcutaneously to the left upper flank of seven male mice. (a) tumors were excised and dissected six weeks later; (b) tumor volume was detected every 3 days after tumor formation; (c) tumor weight was detected. Data represent the mean ± SD of at least triplicate independent experiments; *P < 0.05; **P < 0.01; ***P < 0.001.

To validate the progressive effect of HCG18 in vivo, HN30 cells were stably transfected with HCG18 upregulated lentivirus, and an HCG18-overexpressed xenograft model was constructed (Figure 5a). Our data revealed that the HCG18-overexpressed xenograft had a larger tumor size (Figure 5b) and tumor weight (Figure 5c) compared to the xenograft transfected with control lentivirus. These results confirmed that upregulation of HCG18 promoted the progression of HNSCC cells in vivo.

## Discussion

LncRNAs are considered as crucial regulators in the process of tumorigenesis[15]. Certain lncRNAs including XIST, MALAT, HOTAIR, and LINC00052 have been validated as valuable diagnostic or prognostic biomarkers in HNSCC[16]. The unique signature of lncRNAs in a distinct type of solid tumor makes it possible to provide individualized therapy for HNSCC[17]. These lncRNAs have been shown to be associated with favorable clinical outcomes [18] and thus considered as therapeutic targets of HNSCC[19]. However, much work remains to be done to identify the novel lncRNAs that may participate in the tumorigenesis of HNSCC and investigate the underlying mechanism.

In previous studies, HCG18 was reported to facilitate the progression of gastric cancer, lung adenocarcinoma, and colorectal cancer and indicates poor prognosis of these malignancies[20]. However, the role of HCG18 in HNSCC has not been reported. This study showed that HCG18 expression was elevated in both HNSCC patient tissues and cell lines, and the expression of HCG18 was positively correlated with OS and lymph node metastasis, indicating that HNSCC patients with higher HCG18 expression levels had poorer clinical outcomes. Furthermore, we found that HCG18 accelerated the progression of HNSCC in vitro and in vivo, validating the cellular effect of HCG18 in HNSCC.

There is a growing interest in how HCG18 regulates the biological processes of cancers[21]. In the previous studies, as the most canonical and most reported regulatory pattern of lncRNA [22,23], HCG18 could interact with certain miRNAs to facilitate the progression in solid tumors [24,25]. It was confirmed that silencing of HCG18 could inhibit gastric cancer progression via obstructing the activation of PI3K/Akt signaling[26]. HCG18 could sponge with miR-1271, subsequentially activate MTDH/Wnt/β-catenin pathway, and ultimately fascinate the growth and invasion of colorectal cancer[27]. However, the interaction between HCG18 and functional protein have been rarely reported. In our study, we observed that HCG18 could bind with cyclin D1, a key downstream functional protein of WNT pathway. Cyclin D1 was reported to be overexpressed and indicated poor prognosis in HNSCC patients[28]. We also found that the WNT signaling pathway was also activated by increasing the expression of HCG18, and overexpressed cyclin D1 could reverse the cell effect and WNT-pathway-related proteins. These results indicated that HCG18 could promote HNSCC progression via cyclin D1 and subsequently activate the Wnt signaling pathway. LncRNAs can also competitively bind with miRNAs, resulting in the reduction of gene degradation caused by miRNAs[29]. The other lncRNAs can act with certain functional proteins and regulate downstream signaling[30]. More studies are needed to investigate whether HCG18 interacts with miRNAs that could affect the WNT pathway and promote HNSCC progression.

## Conclusions

The expression of lncRNA HCG18 increased in both HNSCC patient tissues and HNSCC cell lines. Increased expression of HCG18 is positively correlated with the prognosis of HNSCC patients. HCG18 accelerated the proliferation, migration, and invasion of HNSCC cells, and it might exert its regulatory role through cyclin D1 and subsequently activate the WNT signaling pathway. Our results indicate that lncRNA HCG18 might act as a promising prognostic biomarker and a potential therapeutic target in HNSCC.
